# There's Something Wrong with my MAM; the ER–Mitochondria Axis and Neurodegenerative Diseases

**DOI:** 10.1016/j.tins.2016.01.008

**Published:** 2016-03

**Authors:** Sebastien Paillusson, Radu Stoica, Patricia Gomez-Suaga, Dawn H.W. Lau, Sarah Mueller, Tanya Miller, Christopher C.J. Miller

**Affiliations:** 1 Department of Basic and Clinical Neuroscience, Institute of Psychiatry, Psychology and Neuroscience, King's College London, London, SE5 9NU, UK; 2 Churchill Hospital, Old Road, Headington, Oxford, OX3 7LE, UK

**Keywords:** Alzheimer's disease, Parkinson's disease, amyotrophic lateral sclerosis/fronto-temporal dementia, mitochondria associated ER membranes, mitochondria, endoplasmic reticulum

## Abstract

Alzheimer's disease (AD), Parkinson's disease (PD), and amyotrophic lateral sclerosis with associated frontotemporal dementia (ALS/FTD) are major neurodegenerative diseases for which there are no cures. All are characterised by damage to several seemingly disparate cellular processes. The broad nature of this damage makes understanding pathogenic mechanisms and devising new treatments difficult. Can the different damaged functions be linked together in a common disease pathway and which damaged function should be targeted for therapy? Many functions damaged in neurodegenerative diseases are regulated by communications that mitochondria make with a specialised region of the endoplasmic reticulum (ER; mitochondria-associated ER membranes or ‘MAM’). Moreover, several recent studies have shown that disturbances to ER–mitochondria contacts occur in neurodegenerative diseases. Here, we review these findings.

## Trends

Mitochondria and the ER form close physical contacts.

ER–mitochondria contacts regulate functions damaged in neurodegenerative diseases.

ER–mitochondria contacts are damaged in neurodegenerative diseases.

## Alzheimer's Disease, Parkinson's Disease, Amyotrophic Lateral Sclerosis, and Frontotemporal Dementia Are Major Diseases for which We Have No Cures

AD, PD, and ALS/FTD are devastating neurodegenerative diseases that afflict huge numbers of the world's population. It has been estimated that there are 46 million people worldwide with dementia, with AD being the most common form of this diseasei. In addition, more than 7 million people are believed to be living with PDii. ALS is the most common form of motor neuron disease and is estimated to afflict over 400,000 people in the worldiii. Moreover, ALS is now known to be clinically, pathologically, and genetically linked to FTD, which is the second most-common form of presenile dementia after AD [1]. Together, these diseases not only inflict a huge amount of suffering to both patients and carers, but also represent a massive economic burden to our societies: the global cost of dementia alone has recently been estimated to be US$ 818 billion^i^.

Despite these human and economic costs, there are still no cures for AD, PD, or ALS/FTD. Indeed, for AD and ALS/FTD, there are not even effective disease-modifying treatments. Improving and refining current pharmacological approaches represents one route to tackle this problem, but there is also a strong case to be made for identifying and developing new therapeutic targets.

## Convergence of Pathological and Genetic Lesions in AD, PD, and ALS/FTD

Despite affecting different regions of the nervous system, AD, PD, and ALS/FTD share several common features. All involve distinct protein pathologies and, although most cases are sporadic, there are also important genetic components. In AD, the principal pathological features are extracellular accumulations of **amyloid β** (Aβ; see Glossary) peptides within neuritic plaques and intraneuronal accumulations of the microtubule-associated protein **Tau** within **neurofibrillary tangles** 2, 3. Aβ is derived by proteolytic processing from the **amyloid precursor protein** (APP) and mutations in *APP* are causative for some dominantly inherited forms of Alzheimer's disease. Moreover, mutations in the **Presenilin** genes (*PSEN*), which encode key proteins of the γ-secretase enzyme complex involved in cleaving APP to produce Aβ also cause familial AD 2, 3. Many of these *APP* and *PSEN* mutations affect APP processing and Aβ production 2, 3.

In a similar fashion, pathological and genetic evidence converge for PD. This disease is characterised by the degeneration of dopaminergic neurons in the substantia nigra together with intraneuronal inclusions termed ‘Lewy bodies’. α-Synuclein is the major protein constituent of Lewy bodies and genetic abnormalities of *SNCA*, the gene encoding **α-synuclein**, cause dominantly inherited PD [4]. Tau pathology is also seen in some forms of dementia linked with Parkinsonism (**FTD** and Parkinsonism linked to chromosome 17; FTDP-17) and mutations in *MAPT* (the gene encoding Tau) are causative for these disease forms [2].

Likewise, for ALS/FTD, deposits of **Tar DNA-binding protein 43** (TDP-43), **Fused in Sarcoma** (FUS), and **dipeptide repeat proteins** derived from the ***C9ORF72*** gene are major pathologies, and mutations in *TDP43*, *FUS*, and *C9ORF72* cause dominantly inherited forms of disease [1]. Thus, for all of these major neurodegenerative diseases, there is convergence of pathological and genetic evidence for each disease. This has focussed much recent therapeutic work on inhibiting development of the underlying pathologies. However, so far, no drugs targeting these pathologies have entered clinical practice.

## Damage to a Large Number of Cellular Processes Occurs in AD, PD, and ALS/FTD

While there is individual convergence of pathological and genetic evidence for each of the three major neurodegenerative diseases, there is also remarkable similarity in the damage to downstream physiological processes that occurs in all three diseases. Thus, features of all three diseases include damage to mitochondria, Ca^2+^ homeostasis, lipid metabolism, axonal transport, the ER [involving activation of the **unfolded protein response** (UPR)], autophagy, and also inflammatory responses 5, 6, 7, 8, 9, 10, 11, 12, 13, 14, 15, 16. One conundrum is how damage to so many apparently disparate physiological processes might link together in a common disease pathway. Moreover, the diversity of these features also makes devising new treatment strategies difficult. If alternatives to targeting underlying proteinaceous pathologies are to be considered as treatments, then which of these different disrupted cellular functions should be prioritised as a therapeutic target? This point has been eloquently discussed for AD [17].

## Mitochondria Form Close Associations with the ER and these Regulate Many of the Functions that Are Damaged in Neurodegenerative Diseases

Mitochondria have pivotal roles in a variety of cellular functions, including energy metabolism, Ca^2+^ homeostasis, lipid synthesis, and apoptosis. These functions require a dynamic spatial organisation that permits relaying of signals to and from other organelles. In particular, mitochondria are associated with the ER, with 5–20% of the mitochondrial surface closely apposed (10–30 nm distance) to ER membranes 18, 19, 20, 21. These ER membrane domains are known as **MAM**. A large body of evidence demonstrates that mitochondria communicate directly with ER through MAM to regulate several fundamental cellular processes 19, 20, 21. These are discussed below.

### Ca^2+^ Exchange

ER–mitochondria contacts facilitate Ca^2+^ exchange between the two organelles and, in particular, uptake of Ca^2+^ by mitochondria following its release from ER stores via inositol 1,4,5-trisphosphate (IP3) receptors. Ca^2+^ is required by mitochondria for generating ATP via the tricarboxylic acid cycle since several mitochondrial enzymes involved in ATP synthesis (e.g., some dehydrogenases) are regulated by Ca^2+^ 19, 20, 21. The Ca^2+^ concentrations required to elicit a response at the mitochondrial surface are high, but having close contacts with the ER (where luminal concentrations are up to 0.5 mM) means that Ca^2+^ released from the ER can achieve high local concentrations (Ca^2+^ puffs) capable of driving an effect. However, excessive uptake of Ca^2+^ by mitochondria can lead to opening of the mitochondrial permeability transition pore and signalling for apoptosis [19].

### Phospholipid Exchange

ER–mitochondria contacts facilitate phospholipid exchange between the two organelles. This is important because the enzymes involved in some lipid biosynthesis are present in both organelles and so exchange is required for their production 19, 20, 21. Indeed, MAM are now known to be a specialised type of lipid raft (also known as detergent-resistant membranes) [22].

### Intracellular Trafficking

ER–mitochondria associations are linked to intracellular trafficking of mitochondria and ER. Both ER and mitochondria are transported by kinesin 1 and cytoplasmic dynein molecular motors 23, 24. In neurons, kinesin 1 drives anterograde (towards the synapse) transport of mitochondria through axons (axonal transport) [24]. Attachment of mitochondria to kinesin 1 involves the outer mitochondrial membrane protein Miro, which acts as a Ca^2+^ sensor; elevated Ca^2+^ levels halt transport 25, 26, 27. Miro has been shown to localise to ER–mitochondria contact sites and a proportion of ER has been shown to be cotransported with mitochondria 28, 29. Thus, some ER may be cotransported with mitochondria through axons and Miro may sense Ca^2+^ exchange between the two organelles to regulate this transport in response to physiological stimuli.

### ER Stress and the UPR

ER–mitochondria contacts have now been linked to ER stress and the UPR. The ER–UPR is an intracellular signalling pathway that is activated by the accumulation of unfolded proteins in the ER, which then stimulates transcriptional responses to modulate the protein-folding capacity of the ER [9]. Two of the known **tethering proteins** involved in connecting ER with mitochondria, mitofusin 2 and vesicle-associated membrane protein-associated protein B (VAPB) 30, 31, 32, have roles in the UPR 33, 34, 35. Also, a variety of ER chaperones involved in protein folding, such as BiP, calnexin, calreticulin, ERp44, ERp57, and the **Sigma 1 receptor**, are present in MAM [36], and structural uncoupling of ER from mitochondria induces ER stress and the UPR [37]. Thus, crosstalk between ER and mitochondria at ER–mitochondria contacts may have a role in facilitating stress responses and UPR.

### Autophagy

ER–mitochondria associations have been linked to autophagy, a mechanism by which aggregated proteins and damaged organelles are removed from the cytoplasm [5]. Autophagy involves engulfment of damaged proteins and organelles by double-membrane autophagosomes and then fusion of these vesicles with lysosomes to permit degradation of their contents. The source of autophagosomal membranes is not fully known, but some have recently been shown to form at ER–mitochondria contact sites [38].

### Mitochondrial Biogenesis

A role for ER–mitochondria contacts relates to mitochondrial biogenesis. Thus, mitochondrial fission occurs at sites of ER–mitochondria contacts 39, 40 and mitofusin 2, which regulates mitochondrial fusion, is also a proposed tethering protein that connects ER with mitochondria (see below) [30].

### Inflammasome Formation

Finally, ER–mitochondria associations have been linked to formation of the inflammasome. Tissue damage and cell stresses, such as occur in neurodegenerative diseases, are sensed by the innate immune system through pattern recognition receptors. One class of these is the **NOD-like receptors** (NLRs), which sense abnormal cytosolic changes. Upon activation, some NLRs, including NLRP3, form multiprotein complexes, which have been named the ‘**inflammasome**’; these function to initiate proteolytic maturation of the proinflammatory cytokine interleukin 1β [41]. **Reactive oxygen species** (ROS) from mitochondria are one signal for activation of the NLRP3 inflammasome. Recently, ROS was shown to induce relocation of NLRP to MAM, and this may provide a mechanism whereby NLRP senses damage to mitochondria to activate the inflammasome [42].

Remarkably, all of these functions regulated by ER–mitochondria associations are affected in AD, PD, and ALS/FTD. Such links have generated interest in investigating ER–mitochondria associations in these diseases.

## ER–Mitochondria Tethers

**Figure 1:**
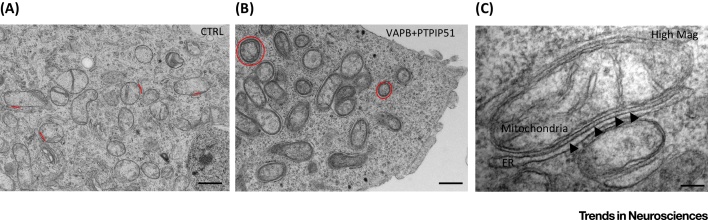
Electron Microscope Images of Endoplasmic Reticulum (ER)-–Mitochondria Contacts in NSC34 Motor Neuron Cells. A control cell is shown (CTRL) (A) along with a cell transfected with the tethering proteins vesicle-associated membrane protein-associated protein B (VAPB) and protein tyrosine phosphatase interacting protein 51 (PTPIP51) (B) [32]. Some selected ER–mitochondria associations are highlighted in red. Transfection of VAPB and PTPIP51 dramatically increases ER–mitochondria associations. (C) A high-magnification image of a mitochondrion with associated ER in a VAPB/PTPIP51 co-transfected cell; putative tethering structures are discernible connecting the two organelles (arrowheads). Scale bars = 500 nm (A,B) and 100 nm (C).

**Figure 2:**
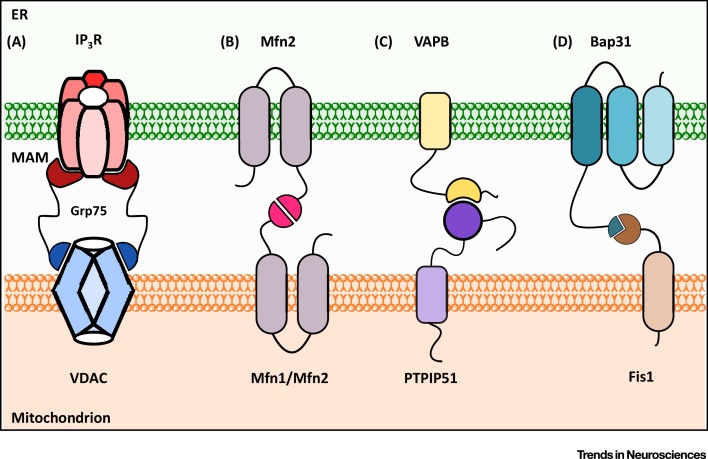
Proposed Endoplasmic Reticulum (ER)–Mitochondria Tethering Protein Complexes. (A) Inositol 1,4,5-trisphosphate (IP3) receptors (IP_3_R) and voltage-dependent anion channel (VDAC) interact via GRP75. (B) ER-located mitofusin 2 interacts with mitochondrial mitofusin1/2 (Mfn1, Mfn2). (C) Vesicle-associated membrane protein-associated protein B (VAPB) binds to protein tyrosine phosphatase interacting protein 51 (PTPIP51). (D) (Bap31) binds to Fission 1 homologue (Fis1).

Despite their fundamental importance to cell metabolism, the mechanisms that mediate recruitment of ER membranes to mitochondria are not fully understood. Electron microscopy (EM) reveals the presence of structures that appear to tether ER with mitochondria [18] (Figure 1), but the biochemical makeup of these is not clear. In yeast, proteins of the ER–mitochondria encounter structure (ERMES) act as a molecular tether between ER and mitochondria [43], but ERMES proteins are yeast specific and no mammalian orthologues have been identified 19, 20, 21. In mammals, interactions between ER-anchored IP3 receptors and the mitochondrial voltage-dependent anion channel (VDAC) via GRP75 were proposed as a tether 44, 45 (Figure 2). However, complete loss of IP3 receptors does not affect ER–mitochondria contacts, which argues against a physical tethering role for these molecules [18]. Homo- and heterotypic interactions between mitochondrial mitofusin 1/2 and ER-located mitofusin 2 have also been proposed as a tethering complex [30] (Figure 2), but later studies from three different laboratories have now shown that loss of mitofusin 2 leads to an increase and not a decrease in ER–mitochondria contacts, which casts doubt on this finding 46, 47, 48. Recently, the integral ER protein VAPB was shown to bind to the outer mitochondrial membrane protein, protein tyrosine phosphatase interacting protein 51 (PTPIP51) to tether ER with mitochondria 31, 32 (Figure 2). VAPB is a MAM protein and binds to PTPIP51 in several different biochemical assays. Modulating VAPB and PTPIP51 expression induces appropriate changes in ER–mitochondria contacts and Ca^2+^ exchange between the two organelles; monitoring Ca^2+^ exchange is a physiological readout of ER–mitochondria associations 31, 32. Recently, the biochemical interaction between VAPB and PTPIP51 was independently verified [49]. Biochemical evidence also links a complex of the mitochondrial fission protein, Fission 1 homologue (Fis1) and ER-located Bap31 with apoptotic functions of ER–mitochondria associations, but the role of these proteins in nonapoptotic processes is unclear [50] (Figure 2). Finally, the multifunctional sorting protein phosphofurin acidic cluster sorting protein 2 (PACS-2) and Bap31 have been linked to ER–mitochondria associations, but whether these are functional scaffolds or regulators of scaffolding protein function are again unclear [37].

Thus, several different protein complexes have been proposed as ER–mitochondria tethers, but since the distances between physiological ER–mitochondria contacts varies between approximately 10 nm and 30 nm 18, 19, 20, 21, it may be that a variety of tethering proteins exists. Indeed, both rough and smooth ER form contacts with mitochondria [18] and, therefore, having different ER-tethering proteins may permit the selective recruitment of different domains and subdomains of ER.

## ER–Mitochondria Associations Are Disrupted in AD, PD, and ALS/FTD

Since many functions regulated by ER–mitochondria associations are damaged in neurodegenerative diseases, several recent studies have investigated ER–mitochondria contacts in these disorders. We now know that ER–mitochondria associations are disrupted in AD, PD, and ALS/FTD 22, 31, 32, 51, 52, 53, 54, 55, 56, 57, 58, 59, 60.

For AD, both Presenilin 1 and Presenilin 2 (major components of the γ-secretase complex that processes APP to release Aβ and which are both mutated in familial AD) are present in MAM [57]. There is much evidence that the AD mutant Presenilins are catalytic loss-of-function mutants [61] and both loss of Presenilins and expression of mutant Presenilins have been shown to affect ER–mitochondria associations and related functions 22, 56, 62. Likewise, alterations to ER–mitochondria associations and functions are seen in APP transgenic mouse models, and treatment of neurons with Aβ affects ER–mitochondria contacts [55]. Also, MAM have been shown to be a site of production of Aβ and this is consistent with the localisation of Presenilins to these regions of ER 57, 63. Finally, the ɛ4 allele of **apolipoprotein E** (ApoE4) has been shown to upregulate the activity of MAM [60]. Individuals carrying one or two copies of the ApoE4 allele have an increased risk of developing AD compared with those carrying the ApoE3 allele [64].

In PD, α-synuclein, **Parkin**, and **protein deglycase** (DJ-1) (which are mutated in different familial forms of disease) all alter ER–mitochondria associations 51, 52, 53, 54. Moreover, α-synuclein, which is most strongly linked to PD, is present in MAM, although its functional role in this compartment is not clear [51].

In ALS/FTD, overexpression of both wild-type and familial ALS/FTD mutant TDP-43 has been shown to not only reduce ER–mitochondria associations, but also disrupt Ca^2+^ exchange between the two organelles, which is a physiological readout of ER–mitochondria contacts [32]. The finding that wild-type as well as mutant TDP-43 decrease ER–mitochondria associations is consistent with the phenotypes observed in TDP-43 transgenic mice, where both wild-type and mutant TDP-43 induce disease [65]. Indeed, there is evidence that mutations in the 3′ untranslated region of *TDP43* cause disease by increasing wild-type TDP-43 protein expression [66]. Likewise, loss of Sigma 1 receptor (which is responsible for some familial forms of ALS/FTD) has been shown to break ER–mitochondria associations [58]. Most recently, **receptor expression enhancing protein 1** (REEP1), which is linked to **hereditary spastic paraplegia** (HSP) and some hereditary motor neuron disorders, has been shown to influence ER–mitochondria associations [59]. REEP1 localises to ER–mitochondria contact sites and, compared with wild type, HSP REEP1 disease mutants have diminished ER–mitochondria associations [59].

While all of the above findings demonstrate defective ER–mitochondria associations in disease, there are some inconsistencies in the different studies. Thus, for AD, one group reported that loss of familial Alzheimer's mutant Presenilin 1 increased ER–mitochondria interactions, whereas another reported that Presenilin 2 but not Presenilin 1 increased these interactions 22, 56. A further study presented evidence that loss of Presenilin 1 function was associated with decreased, not increased ER–mitochondria tethering [62]. Likewise, for PD, one report showed that expression of wild-type and mutant PD α-synuclein decreased, whereas another showed that it increased, ER–mitochondria contacts 51, 53. Also, reports that overexpression of α-synuclein, Parkin, and DJ-1 all increase ER–mitochondria interactions are not simple to reconcile 52, 53, 54. This is because the genetics of PD indicates that overexpression of α-synuclein, but loss of Parkin and DJ-1, cause disease.

The reasons for these different findings are not fully clear, but may relate to the methods used to quantify ER–mitochondria contacts. While assaying biochemical and physiological readouts of ER–mitochondria associations (e.g., Ca^2+^ exchange and phospholipid metabolism) are valid, it is also important to measure how an experimental challenge affects the physical distances between mitochondria and ER, and the proportions of the mitochondrial surface that are apposed to ER. Some studies have utilised confocal microscopy to quantify these parameters, but this does not permit accurate quantification of the 10–30-nm distances that define ER–mitochondria associations 18, 19, 20, 21. Indeed, the identification of mitofusin 2 as an ER–mitochondria tethering protein involved confocal microscopy, but subsequent EM studies (which can reliably quantify 10–30-nm distances) have challenged this finding 30, 46, 47, 48. Others have also highlighted the need for methods that provide higher resolution than confocal microscopy to quantify ER–mitochondria contacts [21]. One possibility is that increases in ER–mitochondria contacts detected by confocal microscopy after exposure to neurodegenerative disease insult represent an artefact due to a redistribution of mitochondria within cells. For example, many neurodegenerative disease insults, such as Aβ and α-synuclein, affect intracellular trafficking of mitochondria (in neurons, this includes axonal transport) to induce accumulation of mitochondria in perinuclear regions, which are regions rich in ER 67, 68. Such mitochondrial accumulation does not necessarily lead to increased physiological communications with ER, and confocal microscopy is unable to accurately detect whether this is the case.

Of course, it may be that different neurodegenerative disease insults affect ER–mitochondria associations in different ways, some increasing and some decreasing contacts. Indeed, abnormal increases and decreases to contacts are both likely to be detrimental to cells. Increasing contacts are predicted to lead to increased mitochondrial Ca^2+^ levels, which could induce opening of the mitochondrial permeability transition pore and signalling for apoptosis [19]. By contrast, reducing contacts may lead to reductions in mitochondrial Ca^2+^ levels and decreased ATP production. Indeed, transgenic APP, Tau, and Presenilin 1 mouse models all display reduced ATP production and elegant recent studies highlighted how even relatively minor reductions in ATP production can induce disease 69, 70. Whatever the precise details, accurate methods to determine how neurodegenerative disease insults affect ER–mitochondria contacts and to correlate any changes with biochemical and physiological readouts of ER–mitochondria associations are required (Box 1).

## The Mechanisms Regulating ER-Mitochondria Contacts and how these Might be Perturbed in Neurodegenerative Diseases

As detailed above, studies to identify new ER–mitochondria tethering proteins are still underway and this has limited how signal transduction and other cellular processes might impact upon ER–mitochondria associations. One attractive notion is that changes in physiological demands of the cell (e.g., requirements for increased mitochondrial ATP or altered lipid production) might somehow signal to induce changes in ER–mitochondria contacts that could facilitate such demands. PACS2 and the mitochondrial ubiquitin ligase MITOL may function in this way, but how they affect tethering proteins remains unclear 37, 71.

Despite these limitations, some of the mechanisms regulating the VAPB–PTPIP51 tethers are now being revealed and these have direct implications for neurodegenerative diseases. **Glycogen synthase kinase 3β** (GSK-3β) has been shown to be a regulator of the VAPB–PTPIP51 interaction; activating reduces whereas inhibiting GSK-3β increases binding of VAPB to PTPIP51 [32]. The precise mechanism underlying this effect is not clear, but may occur via direct phosphorylation of VAPB and/or PTPIP51 by GSK-3β to inhibit their binding, or signalling via GSK-3β to downstream effectors that somehow influences VAPB/PTPIP51 phosphorylation and/or binding.

**Figure 3:**
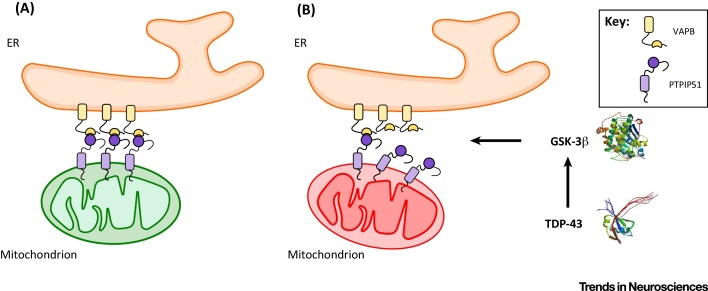
Tar DNA-Binding Protein 43 (TDP-43) Loosens Endoplasmic Reticulum (ER)–Mitochondria Associations in Amyotrophic Lateral Sclerosis with Associated Frontotemporal Dementia (ALS/FTD). (A) Normal situation. (B) Disease situation. TDP-43 induces activation glycogen synthase kinase 3β (GSK-3β), which then disrupts binding of vesicle-associated membrane protein-associated protein B (VAPB) to protein tyrosine phosphatase interacting protein 51 (PTPIP51) to reduce ER–mitochondria associations and Ca^2+^ exchange between the two organelles.

Whatever the route, such findings provide a mechanism to connect TDP-43 and ER–mitochondria tethering in ALS/FTD. TDP-43 induced disruption to ER–mitochondria associations has been shown to involve breaking of the VAPB–PTPIP51 tethers [32]. Moreover, several groups have now reported links between TDP-43 and GSK-3β activity 32, 72, 73, 74. Thus, in disease states, TDP-43 may activate GSK-3β to inhibit binding of VAPB to PTPIP51 and so reduce ER–mitochondria associations (Figure 3). Therefore, activation of GSK-3β may be a key event in TDP-43-linked ALS/FTD. Since GSK-3β is also strongly implicated in AD and PD 75, 76, alterations to GSK-3β activity may be a common mechanism for disrupting the VAPB–PTPIP51 tethers and ER–mitochondria associations in at least some forms of neurodegenerative disease.

## The ER–Mitochondria Axis as a Therapeutic Target

**Figure 4:**
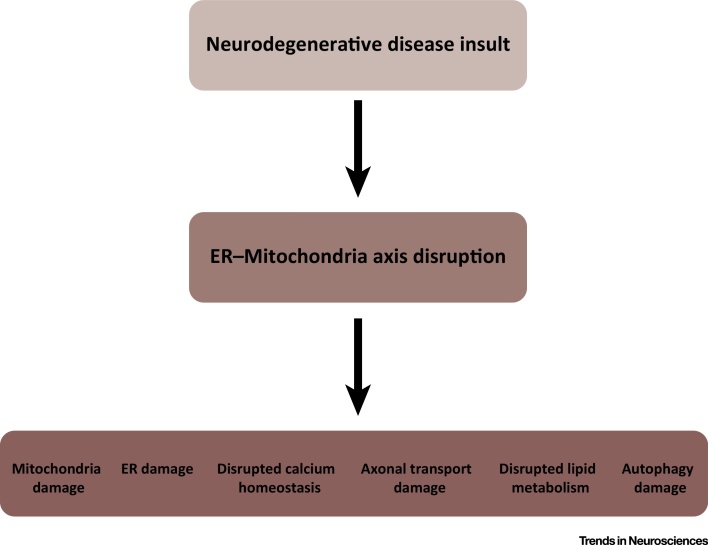
Disruption to Endoplasmic Reticulum (ER)–Mitochondria Associations Provides a Mechanism by which many of the Disparate Pathological Features of Neurodegenerative Diseases Might Arise.

Together, the above findings highlight damage to ER–mitochondria associations as a new pathogenic mechanism in AD, PD, and ALS/FTD. Damage to ER–mitochondria associations also provides an explanation for the seemingly disparate features of these neurodegenerative diseases, since the ER–mitochondria axis regulates many cell functions that are disrupted in disease (Figure 4). Thus, correcting damaged ER–mitochondria associations may correct damage to other neurodegenerative disease-linked features.

For AD and PD, there is not as yet complete consensus on the effects of different disease-associated insults, and the mechanisms leading to altered ER–mitochondria associations are not clear. However, for ALS/FTD, two different insults (TDP-43 and loss of Sigma 1 receptor function) have been shown to loosen ER–mitochondria associations; these studies involved quantification of ER–mitochondria contacts by EM and proximity ligation assays 32, 58. These assays are more capable of detecting changes in ER–mitochondria contacts than other immunocytochemical and confocal microscope-based methods (Box 1). Such findings are in line with a recent elegant study that identified lowered ATP production by mitochondria as driver of motor neuron degeneration in ALS [70]; the TDP-43 and Sigma 1 receptor loss-induced loosening of ER–mitochondria associations are predicted to lower mitochondrial ATP production 18, 19, 20, 21. Moreover, the finding that TDP-43-induced loosening of ER–mitochondria contacts involves breaking of the VAPB–PTPIP51 tethers identifies a molecular target against which to screen potential drugs [32]. Thus, the case for targeting ER–mitochondria contacts as a therapeutic target is strongest for ALS/FTD.

## Concluding Remarks and Future Directions

AD, PD, and ALS/FTD are major diseases for which we have no cures. Although all are classified as neurodegenerative diseases, they are clinically distinct and involve damage to different neuronal populations. Moreover, damage is linked to the development of protein inclusions within affected neurons and the constituent proteins can be different for each disease. Despite these differences, all three diseases display some common features. In particular, they all involve disruption to several seemingly disparate cellular functions, including damage to mitochondria, Ca^2+^ homeostasis, lipid metabolism, axonal transport, the ER/UPR, autophagy, and inflammatory responses. Recently, several studies have shown that some insults associated these neurodegenerative diseases perturb ER–mitochondria associations and linked signalling. ER–mitochondria associations regulate many of the functions that are perturbed in AD, PD, and ALS/FTD. Thus, damage to the ER–mitochondria axis provides a mechanism by which these different disease features might arise.

In future studies, the effects of other neurodegenerative disease insults on the ER–mitochondria axis need to be investigated to determine whether all or only some induce damage and, also, whether any damage is similar for different insults. For PD, it will be important to determine whether mutant genes that cause more common familial forms of PD, such as that encoding **Leucine-rich repeat kinase 2** (*LRRK2*), affect ER–mitochondria associations. There is circumstantial evidence that this may be the case, since most patients with familial LRRK2 display α-synuclein pathology and α-synuclein affects ER–mitochondria contacts 51, 53, 77. Also, LRRK2 has been linked to GSK-3β activity, possibly via the **Wnt signalling pathway** 78, 79, 80 and GSK-3β is a regulator of the VAPB–PTPIP51 interaction [32]. Likewise, for ALS/FTD, the effects of other familial disease genes needs to be investigated, especially ones that are linked more specifically to either ALS or FTD. TDP-43 and Sigma 1 receptor cause both familial ALS and FTD, but some mutant genes such as that encoding mutant **superoxide dismutase 1** (*SOD1*) cause ALS, whereas others, such as *MAPT*, are linked to FTD [1].

For such studies, it will be important to utilise methods that can reliably quantify ER–mitochondria contacts of 10–30-nm distances. As detailed above and in Box 1, confocal microscopy has been used in some studies, but this cannot properly resolve such distances and its use may have led to conflicting results.

Future studies also need to identify more clearly, the mechanisms involved in damage. For example, do different neurodegenerative disease insults that affect ER–mitochondria associations all do so via a common mechanism or might there be different routes for damage. Such information will facilitate any therapeutic targeting of ER–mitochondria associations for neurodegenerative disease (see Outstanding Questions).

Outstanding Questions The identification of the full complement of proteins that directly tether ER and mitochondria in mammals.

Understanding the mechanisms that regulate interactions between tethers so as to control ER–mitochondria associations.

A proper consensus on the effects that different AD, PD, and ALS/FTD insults have on ER–mitochondria associations and tethers.

Understanding the mechanisms by which neurodegenerative disease insults affect ER–mitochondria associations. Do all insults target the VAPB–PTPIP51 tethers and, if so, by what mechanism do they induce damage?

Understanding the mechanisms by which TDP-43 activates GSK-3β and by which activated GSK-3β affects binding of VAPB to PTPIP51. Does this involve direct phosphorylation of VAPB and/or PTPIP51 to disrupt the interaction?

Understanding how the ER–mitochondria axis might be selectively damaged in Ad, PD, and ALS/FTD, since these diseases involve different neuronal populations and different pathogenic proteins.

## Box 1 Methods to Quantify ER–Mitochondria Associations

Physiological contacts between ER and mitochondria are defined as involving distances of approximately 10–30 nm 18, 19, 20, 21. Such close connections are required to properly facilitate Ca^2+^ and phospholipid exchange between the two organelles. Some studies have utilised confocal microscopy to quantify ER–mitochondria contacts. Here, ER and mitochondria are immunostained using different fluorophores and colocalisation of pixels (e.g., via Mander's overlap coefficient) is then used to quantify ER–mitochondria associations. A criticism of this approach is that confocal microscopy does not permit accurate quantification of distances involving 10–30 nm. Indeed, the lateral resolution of the confocal microscope is approximately 250 nm [81], which is about an order of magnitude less than that required. For this reason, other methods need to be used that are capable of properly resolving 10–30-nm distances (e.g., 18, 21, 32). EM is one such method, but here it is important to quantify the proportion of the mitochondrial surface closely apposed to ER and not simply the number of contacts. Thus, a mitochondrion that forms three different contacts with ER that involve only 5% of its surface may well have lower physiological communications with the ER than a mitochondrion that forms a single contact involving over 20% of its surface. Scoring methods need to reflect this aspect.

Super-resolution light microscopy methods, such as structured illumination microscopy (SIM), photoactivation localisation microscopy (PALM), and stochastic optical reconstruction microscopy (STORM), provide alternative nonEM routes for quantifying ER–mitochondria contacts. SIM is probably the simplest to utilise technically and provides lateral resolution of approximately 50 nm 82, 83. STORM and PALM, which involve single-molecule fluorescent localisation methods, provide resolutions of approximately 20 nm laterally and 50 nm axially [83]. Some of these methods can also provide 3D information and data from living cells with up to approximately 100-Hz resolution for SIM [82]. STORM has already been successfully used to analyse ER and mitochondria co-distributions in living cells [84].

Alternative microscopy methods include use of *in situ* proximity ligation assays [85]. Here, conventionally fixed cells and tissues are probed with primary antibodies to ER and outer mitochondrial membrane proteins that are known to reside in close proximity (e.g., tethering proteins), followed by secondary antibodies coupled to specific oligonucleotides. These oligonucleotides facilitate hybridisation and ligation of connector oligonucleotides to form a circular DNA molecule, which then serves as a template for rolling circular amplification. Use of labelled nucleotides enables microscopic detection and quantification of hybridisation signals. The distances detected by proximity ligation assays are similar to those detected by resonance energy transfer between fluorophores (i.e., approximately 30 nm) [85]. Such proximity ligations assays have already been used to quantify ER–mitochondria associations 55, 58 and binding of VAPB to PTPIP51 [31]. Other methods include use of dimer-dependent fluorophores fused to proteins that interact at the ER–mitochondria interface, although the effects of expression *per se* of such proteins on ER–mitochondria contacts may need to be taken into account in any quantification [86].

Finally, functional assays of ER–mitochondria associations, such as lipid metabolism and/or Ca^2+^ homeostasis, can be used as complementary physiological readouts. Temporal measurements of Ca^2+^ uptake by mitochondria following physiological release from ER stores are particularly relevant in this context, since tightening and loosening of contacts are predicted to alter the times required for peak mitochondrial Ca^2+^ uptake 31, 87.

## Glossary

Amyloid β (Aβ): an approximate 40–43 amino acid peptide that is the major protein constituent at the core of the **amyloid plaque**. The longer forms of Aβ are believed to be pathogenic.
Amyloid plaque: regions of degenerating neurites surrounding an amyloid (Aβ) core. A hallmark pathology of AD.
Amyloid precursor protein (APP): a type-1 membrane-spanning protein from which Aβ peptides are derived. APP is cleaved by β- and γ-secretases to release Aβ. The *APP* gene is mutated in some familial forms of AD and some of these mutations affect APP processing and Aβ production. Duplication of the *APP* gene also causes familial AD.
Apolipoprotein E (ApoE): functions to mediate lipid transport between tissues and/or cells. There are three major APOE alleles corresponding to combinations of amino acids at residues 112 and 158 (E2, E3, and E4). The APOE4 allele is the major genetic risk factor for late-onset AD.
C9ORF72 (and dipeptide repeat proteins): mutations in the *C9ORF72* gene cause a large number of familial ALS/FTD cases. The disease-causing mutations involve expansion of a hexanucleotide repeat within intron 1, but how this causes disease is not clear. One suggestion is that the expansion reduces expression of C9ORF72, which then leads to disease. Another is that it acts to sequester mRNA-binding proteins and/or transcription factors, such that expression of key genes is disrupted to induce disease. Finally, translation of the intronic repeat has been demonstrated, which produces polypeptides comprising repeating di-amino acids; these dipeptide repeats are a pathology of ALS/FTD.
ER–mitochondria tethering proteins: (i) Fission 1 homologue (Fis1) and Bap31. Mitochondrial Fis1 and ER-located Bap31 are proposed ER–mitochondria tethering proteins linked to apoptosis; (ii) inositol 1,4,5-trisphosphate (IP3) receptors and the mitochondrial voltage-dependent anion channel (VDAC) interact via GRP75 to form a proposed ER–mitochondria tethering complex; (iii) Mitofusin 1 and 2. Mitochondrial proteins involved in mitochondria fusion. Some mitofusin 2 is reported to locate to ER, where it can interact with mitochondrial mitofusin 1/2 to tether the two organelles; (iv) Vesicle-associated membrane protein-associated protein B (VAPB) and protein tyrosine phosphatase-interacting protein 51 (PTPIP51). VAPB is an ER protein that binds to the outer mitochondrial membrane protein PTPIP51. VAPB and PTPIP51 tether ER with mitochondria and these tethers are broken in ALS/FTD. No PTPIP51 homologues are present in *Drosophila melanogaster*, *Caenorhabditis elegans*, or *Saccharomyces cerevisiae*; thus, the VAPB–PTPIP51 tethering complex appears vertebrate specific.
Frontotemporal dementia (FTD): the most common form of presenile dementia after AD.
Fused in sarcoma (FUS): accumulations of FUS are a pathology in some forms of ALS and associated FTD. Mutations in the *FUS* gene cause some familial forms of ALS/FTD.
Glycogen synthase kinase-3β (GSK-3β): a serine/threonine kinase that is strongly linked to several neurodegenerative diseases.
Hereditary spastic paraplegia (HSP): a group of inherited diseases that display clinical features of progressive weakness and spasticity mainly in the lower extremities owing to axonal degeneration.
Leucine-rich repeat kinase 2 (LRRK2): mutations in *LRRK2* are the most common genetic cause of PD.
Lewy bodies: the hallmark, intraneuronal pathology of PD.
Mitochondria-associated membranes (MAM): the regions of ER that are closely associated with mitochondria. A large body of evidence demonstrates that mitochondria communicate with ER via MAM to regulate several fundamental cellular functions.
Neurofibrillary tangles: an intraneuronal hallmark pathology of AD. Under the electron microscope, neurofibrillary tangles comprise bundles of paired helical filaments that are assembled from Tau.
Nod-like receptor protein 3 (NLRP3) and the inflammasome: NLRP3 is a component of the inflammasome, which detects cellular stresses to activate the inflammatory cytokine interleukin 1β.
Parkin: component of the E3 ubiquitin ligase complex involved in targeting of proteins for degradation by the proteasome. Mutations in the gene encoding Parkin cause some forms of autosomal recessive juvenile PD.
Presenilin 1 and 2: components of the γ-secretase enzyme complex involved in cleaving APP to release Aβ. Mutations in the genes encoding Presenilin cause some familial forms of AD. Most of these mutants are ‘loss of function’, but lead to an increase in the relative proportions of Aβ(1–42)/Aβ(1–40).
Protein deglycase (DJ-1): mutations in DJ-1 cause some autosomal recessive early-onset forms of PD.
Reactive oxygen species (ROS): chemically reactive oxygen species that can have damaging effects on cells.
Receptor expression-enhancing protein 1 (REEP1): protein located in MAM and which is mutated in HSP and some distal hereditary motor neuropathies.
Sigma 1 receptor: an ER-resident protein chaperone that is present in MAM. Mutations in the gene encoding Sigma 1 that are believed to be loss of function cause some familial forms of ALS/FTD.
Superoxide dismutase 1 (SOD1): a major antioxidant enzyme. Mutations in *SOD1* cause some dominantly inherited forms of familial ALS.
α-Synuclein: the major protein constituent of Lewy bodies. Point mutations and genomic multiplications of the gene encoding α-synuclein cause some dominantly inherited forms of familial PD.
Tar DNA-binding proteins-43 (TDP-43): accumulations of TDP-43 are a major pathology in ALS and FTD. Mutations in the *TDP43* gene cause some familial forms of ALS/FTD.
Tau: a microtubule-associated protein that is the major biochemical component of paired helical filaments in AD. Mutations in *MAPT* cause familial FTD and Parkinsonism linked to chromosome 17.
Unfolded protein response (UPR): a physiological response of the ER to stresses induced by disturbances of protein folding.
Wnt signalling pathway: a signal transduction pathway that leads to changes in gene expression and which involves GSK-3β.

## Resources

^i^ www.alz.co.uk/research/WorldAlzheimerReport2015.pdf

^ii^ www.pdf.org/en/parkinson_statistics

^iii^ www.alsmndalliance.org

## References

[1] Ling S.C. (2013). Converging mechanisms in ALS and FTD: disrupted RNA and protein homeostasis. Neuron.

[2] Goedert M., Spillantini M.G. (2006). A century of Alzheimer's disease. Science.

[3] Hardy J. (2006). A hundred years of Alzheimer's disease research. Neuron.

[4] Cookson M.R., Bandmann O. (2010). Parkinson's disease: insights from pathways. Hum. Mol. Genet..

[5] Harris H., Rubinsztein D.C. (2011). Control of autophagy as a therapy for neurodegenerative disease. Nat. Rev. Neurol..

[6] Johri A., Beal M.F. (2012). Mitochondrial dysfunction in neurodegenerative diseases. J. Pharmacol. Exp. Ther..

[7] Millecamps S., Julien J.P. (2013). Axonal transport deficits and neurodegenerative diseases. Nat. Rev. Neurosci..

[8] Roussel B.D. (2013). Endoplasmic reticulum dysfunction in neurological disease. Lancet Neurol..

[9] Bernales S. (2012). Unfolded protein stress in the endoplasmic reticulum and mitochondria: a role in neurodegeneration. Front. Aging Neurosci..

[10] Martin M.G. (2014). Cholesterol in brain disease: sometimes determinant and frequently implicated. EMBO Rep..

[11] Schmitt F. (2014). A plural role for lipids in motor neuron diseases: energy, signaling and structure. Front. Cell. Neurosci..

[12] Stutzmann G.E., Mattson M.P. (2011). Endoplasmic reticulum Ca(2+) handling in excitable cells in health and disease. Pharmacol. Rev..

[13] Ferraiuolo L. (2011). Molecular pathways of motor neuron injury in amyotrophic lateral sclerosis. Nat. Rev. Neurol..

[14] Glass C.K. (2010). Mechanisms underlying inflammation in neurodegeneration. Cell.

[15] Hunn B.H. (2015). Impaired intracellular trafficking defines early Parkinson's disease. Trends Neurosci..

[16] Gibbs K.L. (2015). Regulation of axonal transport by protein kinases. Trends Biochem. Sci..

[17] Herrup K. (2015). The case for rejecting the amyloid cascade hypothesis. Nat. Neurosci..

[18] Csordas G. (2006). Structural and functional features and significance of the physical linkage between ER and mitochondria. J. Cell Biol..

[19] Rowland A.A., Voeltz G.K. (2012). Endoplasmic reticulum-mitochondria contacts: function of the junction. Nat. Rev. Mol. Cell Biol..

[20] van Vliet A. (2014). New functions of mitochondria associated membranes in cellular signalling. Biochim. Biophys. Acta.

[21] Helle S.C. (2013). Organization and function of membrane contact sites. Biochim. Biophys. Acta.

[22] Area-Gomez E. (2012). Upregulated function of mitochondria-associated ER membranes in Alzheimer disease. EMBO. J..

[23] Wozniak M.J. (2009). Role of kinesin-1 and cytoplasmic dynein in endoplasmic reticulum movement in VERO cells. J. Cell Sci..

[24] Saxton W.M., Hollenbeck P.J. (2012). The axonal transport of mitochondria. J. Cell Sci..

[25] Macaskill A.F. (2009). Miro1 is a calcium sensor for glutamate receptor-dependent localization of mitochondria at synapses. Neuron.

[26] Saotome M. (2008). Bidirectional Ca2+-dependent control of mitochondrial dynamics by the Miro GTPase. Proc. Natl. Acad. Sci. U.S.A..

[27] Wang X., Schwarz T.L. (2009). The mechanism of Ca2+ -dependent regulation of kinesin-mediated mitochondrial motility. Cell.

[28] Friedman J.R. (2010). ER sliding dynamics and ER-mitochondrial contacts occur on acetylated microtubules. J. Cell Biol..

[29] Kornmann B. (2011). The conserved GTPase Gem1 regulates endoplasmic reticulum-mitochondria connections. Proc. Natl. Acad. Sci. U.S.A..

[30] de Brito O.M., Scorrano L. (2008). Mitofusin 2 tethers endoplasmic reticulum to mitochondria. Nature.

[31] De Vos K.J. (2012). VAPB interacts with the mitochondrial protein PTPIP51 to regulate calcium homeostasis. Hum. Mol. Genet..

[32] Stoica R. (2014). ER-mitochondria associations are regulated by the VAPB-PTPIP51 interaction and are disrupted by ALS/FTD-associated TDP-43. Nat. Commun..

[33] Gkogkas C. (2008). VAPB interacts with and modulates the activity of ATF6. Hum. Mol. Genet..

[34] Kanekura K. (2006). Characterization of amyotrophic lateral sclerosis-linked P56S mutation of vesicle-associated membrane protein-associated protein B (VAPB/ALS8). J. Biol. Chem..

[35] Munoz J.P. (2013). Mfn2 modulates the UPR and mitochondrial function via repression of PERK. EMBO J..

[36] Hayashi T. (2009). MAM: more than just a housekeeper. Trends Cell Biol..

[37] Simmen T. (2005). PACS-2 controls endoplasmic reticulum-mitochondria communication and Bid-mediated apoptosis. EMBO J..

[38] Hamasaki M. (2013). Autophagosomes form at ER-mitochondria contact sites. Nature.

[39] Korobova F. (2013). An actin-dependent step in mitochondrial fission mediated by the ER-associated formin INF2. Science.

[40] Friedman J.R. (2011). ER tubules mark sites of mitochondrial division. Science.

[41] Gross O. (2011). The inflammasome: an integrated view. Immunol. Rev..

[42] Zhou R. (2011). A role for mitochondria in NLRP3 inflammasome activation. Nature.

[43] Kornmann B. (2009). An ER-mitochondria tethering complex revealed by a synthetic biology screen. Science.

[44] Rapizzi E. (2002). Recombinant expression of the voltage-dependent anion channel enhances the transfer of Ca2+ microdomains to mitochondria. J. Cell Biol..

[45] Szabadkai G. (2006). Chaperone-mediated coupling of endoplasmic reticulum and mitochondrial Ca2+ channels. J. Cell Biol..

[46] Cosson P. (2012). Mitofusin-2 independent juxtaposition of endoplasmic reticulum and mitochondria: an ultrastructural study. PLoS ONE.

[47] Filadi R. (2015). Mitofusin 2 ablation increases endoplasmic reticulum-mitochondria coupling. Proc. Natl. Acad. Sci. U.S.A..

[48] Wang P.T. (2015). Distinct mechanisms controlling rough and smooth endoplasmic reticulum-mitochondria contacts. J. Cell Sci..

[49] Huttlin E.L. (2015). The BioPlex network: a systematic exploration of the human interactome. Cell.

[50] Iwasawa R. (2011). Fis1 and Bap31 bridge the mitochondria-ER interface to establish a platform for apoptosis induction. EMBO J..

[51] Guardia-Laguarta C. (2014). alpha-Synuclein is localized to mitochondria-associated ER membranes. J. Neurosci..

[52] Ottolini D. (2013). The Parkinson disease related protein DJ-1 counteracts mitochondrial impairment induced by the tumor suppressor protein p53 by enhancing endoplasmic reticulum-mitochondria tethering. Hum. Mol. Genet..

[53] Cali T. (2012). Alpha-synuclein controls mitochondrial calcium homeostasis by enhancing endoplasmic reticulum-mitochondria interactions. J. Biol. Chem..

[54] Cali T. (2013). Enhanced parkin levels favor ER-mitochondria crosstalk and guarantee Ca(2+) transfer to sustain cell bioenergetics. Biochim. Biophys. Acta.

[55] Hedskog L. (2013). Modulation of the endoplasmic reticulum-mitochondria interface in Alzheimer's disease and related models. Proc. Natl. Acad. Sci. U.S.A..

[56] Zampese E. (2011). Presenilin 2 modulates endoplasmic reticulum (ER)-mitochondria interactions and Ca2+ cross-talk. Proc. Natl. Acad. Sci. U.S.A..

[57] Area-Gomez E. (2009). Presenilins are enriched in endoplasmic reticulum membranes associated with mitochondria. Am. J. Pathol..

[58] Bernard-Marissal N. (2015). Dysfunction in endoplasmic reticulum-mitochondria crosstalk underlies SIGMAR1 loss of function mediated motor neuron degeneration. Brain.

[59] Lim Y. (2015). Hereditary spastic paraplegia-linked REEP1 modulates endoplasmic reticulum/mitochondria contacts. Ann. Neurol..

[60] Tambini M.D. (2015). ApoE4 upregulates the activity of mitochondria-associated ER membranes. EMBO Rep..

[61] De Strooper B. (2007). Loss-of-function presenilin mutations in Alzheimer disease. Talking Point on the role of presenilin mutations in Alzheimer disease. EMBO Rep..

[62] Sepulveda-Falla D. (2014). Familial Alzheimer's disease-associated presenilin-1 alters cerebellar activity and calcium homeostasis. J. Clin. Invest..

[63] Schreiner B. (2015). Amyloid-beta peptides are generated in mitochondria-associated endoplasmic reticulum membranes. J. Alzheimers Dis..

[64] Liu C.C. (2013). Apolipoprotein E and Alzheimer disease: risk, mechanisms and therapy. Nat. Rev. Neurol..

[65] Tsao W. (2012). Rodent models of TDP-43: recent advances. Brain Res..

[66] Gitcho M.A. (2009). TARDBP 3′-UTR variant in autopsy-confirmed frontotemporal lobar degeneration with TDP-43 proteinopathy. Acta Neuropathol..

[67] Nakamura K. (2011). Direct membrane association drives mitochondrial fission by the Parkinson disease-associated protein alpha-synuclein. J. Biol. Chem..

[68] De Vos K.J. (2008). Role of axonal transport in neurodegenerative diseases. Annu. Rev. Neurosci..

[69] Eckert A. (2010). Convergence of amyloid-beta and tau pathologies on mitochondria in vivo. Mol. Neurobiol..

[70] Le Masson G. (2014). A computational model of motor neuron degeneration. Neuron.

[71] Sugiura A. (2013). MITOL Regulates endoplasmic reticulum-mitochondria contacts via Mitofusin2. Mol. Cell.

[72] Ambegaokar S.S., Jackson G.R. (2011). Functional genomic screen and network analysis reveal novel modifiers of tauopathy dissociated from tau phosphorylation. Hum. Mol. Genet..

[73] Sreedharan J. (2015). Age-dependent TDP-43-mediated motor neuron degeneration requires GSK3, hat-trick, and xmas-2. Curr. Biol..

[74] Moujalled D. (2013). Kinase inhibitor screening identifies cyclin-dependent kinases and glycogen synthase kinase 3 as potential modulators of TDP-43 cytosolic accumulation during cell stress. PLoS ONE.

[75] Llorens-Martin M. (2014). GSK-3beta, a pivotal kinase in Alzheimer disease. Front. Mol. Neurosci..

[76] Golpich M. (2015). Glycogen synthase kinase-3 beta (GSK-3beta) signaling: implications for Parkinson's disease. Pharmacol. Res..

[77] Poulopoulos M. (2012). The neuropathology of genetic Parkinson's disease. Mov. Disord..

[78] Lin C.H. (2010). LRRK2 G2019S mutation induces dendrite degeneration through mislocalization and phosphorylation of tau by recruiting autoactivated GSK3beta. J. Neurosci..

[79] Kawakami F. (2014). Leucine-rich repeat kinase 2 regulates tau phosphorylation through direct activation of glycogen synthase kinase-3beta. FEBS J..

[80] Sancho R.M. (2009). Mutations in the LRRK2 Roc-COR tandem domain link Parkinson's disease to Wnt signalling pathways. Hum. Mol. Genet..

[81] Lidke D.S., Lidke K.A. (2012). Advances in high-resolution imaging: techniques for three-dimensional imaging of cellular structures. J. Cell Sci..

[82] Hirano Y. (2015). Recent advancements in structured-illumination microscopy toward live-cell imaging. Microscopy.

[83] Fornasiero E.F., Opazo F. (2015). Super-resolution imaging for cell biologists: concepts, applications, current challenges and developments. Bioessays.

[84] Shim S.H. (2012). Super-resolution fluorescence imaging of organelles in live cells with photoswitchable membrane probes. Proc. Natl. Acad. Sci. U.S.A..

[85] Soderberg O. (2006). Direct observation of individual endogenous protein complexes in situ by proximity ligation. Nat. Methods.

[86] Alford S.C. (2012). Dimerization-dependent green and yellow fluorescent proteins. ACS Synth. Biol..

[87] Csordas G. (2010). Imaging interorganelle contacts and local calcium dynamics at the ER-mitochondrial interface. Mol. Cell.

